# Potential of E-Learning Interventions and Artificial Intelligence–Assisted Contouring Skills in Radiotherapy: The ELAISA Study

**DOI:** 10.1200/GO.24.00173

**Published:** 2024-09-05

**Authors:** Mathis Ersted Rasmussen, Kamal Akbarov, Egor Titovich, Jasper Albertus Nijkamp, Wouter Van Elmpt, Hanne Primdahl, Pernille Lassen, Jon Cacicedo, Lisbeth Cordero-Mendez, A.F.M. Kamal Uddin, Ahmed Mohamed, Ben Prajogi, Kartika Erida Brohet, Catherine Nyongesa, Darejan Lomidze, Gisupnikha Prasiko, Gustavo Ferraris, Humera Mahmood, Igor Stojkovski, Isa Isayev, Issa Mohamad, Leivon Shirley, Lotfi Kochbati, Ludmila Eftodiev, Maksim Piatkevich, Maria Matilde Bonilla Jara, Orges Spahiu, Rakhat Aralbayev, Raushan Zakirova, Sandya Subramaniam, Solomon Kibudde, Uranchimeg Tsegmed, Stine Sofia Korreman, Jesper Grau Eriksen

**Affiliations:** ^1^ Experimental Clinical Oncology, Aarhus University Hospital, Aarhus, Denmark; ^2^ International Atomic Energy Agency, Vienna, Austria; ^3^ Department of Clinical Medicine, Aarhus University, Aarhus, Denmark; ^4^ MAASTRO clinic, Maastricht University Medical Centre, Maastricht, the Netherlands; ^5^ Department of Oncology, Aarhus University Hospital, Aarhus, Denmark; ^6^ Department of Radiation Oncology, Cruces University Hospital, Bilbao, Spain; ^7^ Labaid Cancer Hospital and Super Speciality Centre, Dhaka, Bangladesh; ^8^ National Cancer Institute, University of Gezira, Wad Madani, Sudan; ^9^ Cipto Mangunkusumo Hospital, Jakarta, Indonesia; ^10^ Dharmais Cancer Hospital, Jakarta, Indonesia; ^11^ Kenyatta National Hospital, Nairobi, Kenya; ^12^ Tbilisi State Medical University and Ingorokva High Medical Technology University Clinic, Tbilisi, Georgia; ^13^ Nepal Cancer Hospital and Research Center, Lalitpur, Nepal; ^14^ Centro de Radioterapiya dean Funes, Cordoba, Argentina; ^15^ Atomic Energy Cancer Hospital NORI, Islamabad, Pakistan; ^16^ University Clinic of Radiotherapy and Oncology, Skopje, Macedonia; ^17^ National Center of Oncology, Baku, Azerbaijan; ^18^ King Hussein Cancer Center, Amman, Jordan; ^19^ Christian Institute of Health Science and Research, Dimapur, India; ^20^ Hospital Abderrahmen Mami, Ariana, Tunesia; ^21^ Moldavian Oncology Institute, Chisinau, Moldova; ^22^ N. N. Alexandrov National Cancer Centre of Belarus, Minsk, Belarus; ^23^ Hospital México, San José, Costa Rica; ^24^ Mother Tereza Hospital, Tirana, Albania; ^25^ National Centre of Oncology and Hematology, Bishkek, Kyrgyzstan; ^26^ Center of Nuclear Medicine and Oncology, Semey, Kazakhstan; ^27^ Hospital Kuala Lumpur, Kuala Lumpur, Malaysia; ^28^ Uganda Cancer Institute, Kampala, Uganda; ^29^ National Cancer Center of Mongolia, Ulaanbaatar, Mongolia

## Abstract

**PURPOSE:**

Most research on artificial intelligence–based auto-contouring as template (AI-assisted contouring) for organs-at-risk (OARs) stem from high-income countries. The effect and safety are, however, likely to depend on local factors. This study aimed to investigate the effects of AI-assisted contouring and teaching on contouring time and contour quality among radiation oncologists (ROs) working in low- and middle-income countries (LMICs).

**MATERIALS AND METHODS:**

Ninety-seven ROs were randomly assigned to either manual or AI-assisted contouring of eight OARs for two head-and-neck cancer cases with an in-between teaching session on contouring guidelines. Thereby, the effect of teaching (yes/no) and AI-assisted contouring (yes/no) was quantified. Second, ROs completed short-term and long-term follow-up cases all using AI assistance. Contour quality was quantified with Dice Similarity Coefficient (DSC) between ROs' contours and expert consensus contours. Groups were compared using absolute differences in medians with 95% CIs.

**RESULTS:**

AI-assisted contouring without previous teaching increased absolute DSC for optic nerve (by 0.05 [0.01; 0.10]), oral cavity (0.10 [0.06; 0.13]), parotid (0.07 [0.05; 0.12]), spinal cord (0.04 [0.01; 0.06]), and mandible (0.02 [0.01; 0.03]). Contouring time decreased for brain stem (–1.41 [–2.44; –0.25]), mandible (–6.60 [–8.09; –3.35]), optic nerve (–0.19 [–0.47; –0.02]), parotid (–1.80 [–2.66; –0.32]), and thyroid (–1.03 [–2.18; –0.05]). Without AI-assisted contouring, teaching increased DSC for oral cavity (0.05 [0.01; 0.09]) and thyroid (0.04 [0.02; 0.07]), and contouring time increased for mandible (2.36 [–0.51; 5.14]), oral cavity (1.42 [–0.08; 4.14]), and thyroid (1.60 [–0.04; 2.22]).

**CONCLUSION:**

The study suggested that AI-assisted contouring is safe and beneficial to ROs working in LMICs. Prospective clinical trials on AI-assisted contouring should, however, be conducted upon clinical implementation to confirm the effects.

## Teaser

AI improves contouring quality and saves time for oncologists in low- and middle-income countries.

## BACKGROUND

Radiotherapy is a cornerstone in the treatment of cancer, with an estimated 50% of all patients with cancer needing radiotherapy at some point.^1^ Radiotherapy has been found to be cost-effective across a variety of cancer types and sites and treatment regimens.^2-6^ Despite these benefits, the utilization of radiotherapy has been found to be suboptimal across high-, middle-, and low-income countries globally.^1,7,8^ Although there are many reasons for this, a major contributing factor is lack of trained clinical staff.^1,9^ As the cancer incidence is expected to double in the coming decades,^1^ there is an urgent need to develop and implement tools and strategies to reduce the patient-specific workload. If the development of radiotherapy does not keep pace with the increasing demands, it may lead to further underutilization globally, which inevitably will harm patients.

**CONTEXT**

**Key Objective**
How does teaching and artificial intelligence (AI)–assisted contouring affect contouring quality and time in a global cohort of radiation oncologists working in low- and middle-income countries (LMICs) contouring organs-at-risk (OARs) for head-and-neck cancer?
**Knowledge Generated**
AI-assisted contouring increased contouring quality compared with expert consensus contours regardless of whether teaching was received or not. Teaching increased contouring quality for only two OARs, but increased the time-saving effect of AI-assisted contouring.
**Relevance**
AI-assisted contouring in combination with teaching of contouring guidelines is an effective strategy to reduce contouring time and conform contouring practices within and between radiotherapy departments located in LMIC.

Contouring is a time-consuming task for clinical staff^10^ and is prone to observer variability,^11,12^ but it is essential in modern radiotherapy. Auto-contouring has been studied intensely^11^ and is known to reduce contouring time^13,14^ and interobserver variation^15-19^ across a variety of cancer sites, although manual editing is still required.^20^ Today, auto-contouring is often artificial intelligence (AI)–based and is usually integrated into the clinical workflow as templates for contouring (AI-assisted contouring). AI-assisted contouring may serve as a part of the solution to the underutilization of radiotherapy by reducing the manual workload for clinical staff and thereby reducing the diagnosis-to-treatment times, which may improve patient outcome.^21^ Most research on AI-assisted contouring, however, stem from high-income countries. This is a challenge in low- and middle-income countries (LMICs) since local factors such as patient abundance, clinical and financial resources, technical expertise, and mindsets of clinicians possibly influence how AI-assisted contouring is used. Hence, AI-assisted contouring should be evaluated in the context of LMICs to secure a safe and beneficial implementation worldwide.

The purpose of this study was to investigate how contouring quality and contouring time were affected among radiation oncologists (ROs) working in LMICs by (1) a single teaching session on contouring guidelines and AI-assisted contouring and (2) having AI-assisted contouring available. Subsequently, the effect of teaching (1) combined with AI-assisted contouring (2) was investigated after 2-week and 6-month follow-up periods. The study was a collaboration between the International Atomic Energy Agency (IAEA) and Aarhus University Hospital, Denmark.

## MATERIALS AND METHODS

### Institutions and Participants

Radiotherapy institutions were selected and enrolled by the IAEA according to the following criteria:

1. Located in an LMIC
2. Treating at least 20 patients with head-and-neck cancer per year
3. Performing computed tomography (CT)–based intensity-modulated radiotherapy
4. Able to enroll at least three ROs
5. Access to stable internet connection

Each institution appointed one chief scientific investigator who was in charge of recruiting ROs from the institution. There were no requirements for the ROs except they should have undergone training in head-and-neck contouring. The ROs completed a questionnaire regarding professional background and knowledge about AI-assisted contouring (Data Supplement, Fig S1). As a result, 23 radiotherapy institutions with 97 ROs were enrolled.

### Case Selection

The aim was to have as many patient cases as possible while having at least seven contour sets made per case. On the basis of the experience from previous studies by IAEA, the expected participant dropout rate was 50%. Therefore, optimally, 14 participants should be assigned to each case. Given the study design (see below) and the number of enrolled institutions and participants, this required 16 head-and-neck cancer cases that were provided by Aarhus University Hospital (Data Supplement, Table S1).

### Study Design and Random Assignment

Institutions (including their participants) were randomly assigned to either the control group or the intervention group. Random assignment was balanced on (1) institutions' annual number of patients with head-and-neck cancer and (2) whether any form of auto-contouring was available at the institution.

**FIG 1:**
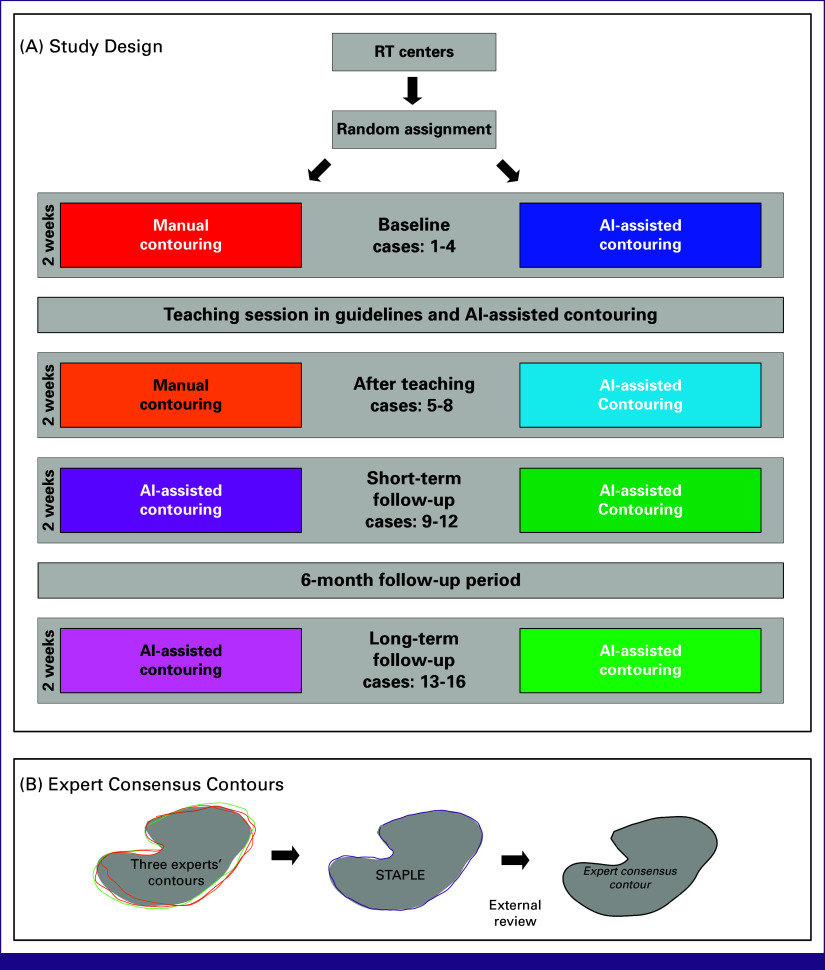
(A) Overview of the study design. The left column (red, orange, dark purple, and light purple) represents the control group, and the right column (dark blue, light blue, dark green, and light green) represents the intervention group. Inside the colored boxes is the type of contouring used by the given study group in the specific round. Contouring rounds are marked as horizontal gray boxes, and the same four cases are used for both study groups inside these. (B) Overview of the method for obtaining expert consensus contours. (1) Three experts contoured all cases and structures independently, (2) the three contour sets were merged with the STAPLE algorithm and binary thresholded at 0.8, and (3) the final STAPLEd structures were reviewed and edited for artifact by one of the initial contourers in consensus with an external head-and-neck expert oncologist. AI, artificial intelligence; RT, radiotherapy; STAPLE, Simultaneous Truth and Performance Evaluation.

With 2 weeks to complete each round, participants were asked to contour eight organs-at-risk (OARs) on one case in each of the four sequential rounds (Fig 1):

1. Before the teaching session (baseline)
2. Immediately after the teaching session (after teaching)
3. Two weeks after the teaching session (short-term follow-up)
4. Six months after short-term follow-up (long-term follow-up)

The control group contoured manually at baseline and after teaching and did AI-assisted contouring in the short-term and long-term follow-ups. The intervention group used AI-assisted contouring in all four rounds. In each round, four new patient cases were used (same cases in both groups). The cases were assigned randomly institution-wise, which resulted in 2-3 institutions (7-15 ROs) in each study group per case.

The effects of teaching and AI-assisted contouring were quantified in a two-by-two fashion with results at baseline and after teaching. To investigate whether the effects of teaching combined with AI-assisted contouring persisted over time, the short-term and long-term follow-ups were compared with the round after teaching within each group.

### Contouring

Contouring took place in EduCase (RadOnc eLearning Center, Inc, Jackson, WY), and the AI-contours were generated by EduCase professionals using Contour+, Guideline-Based Segmentation Solution (MVision AI Oy, Helsinki, Finland), which is based on well-defined contouring guidelines for brain stem^22^ and for the spinal cord, oral cavity, mandible, right submandibular gland, right parotid gland, right optic nerve, and thyroid.^23^ To imitate the clinical reality of many LMICs, only CT scans were available to the participants. Participants were instructed to generate clinically acceptable contours in accordance with the contouring guidelines. If deemed necessary, participants were allowed to delete AI contours and start over with manual contouring. The participants did not have access to the contours of others. Contours were handed in individually, but collaboration between participants was, however, not explicitly disallowed.

### Preprocessing of Contours

Digital Imaging and Communications in Medicine Structure Sets were exported from EduCase and converted to Neuroimaging Informatics Technology Initiative files with a voxel spacing of *x* = 0.39 mm, *y* = 0.39 mm, and *z* = 2 mm (a scaling factor in X and Y of 3 times the CT grid).^24,25^

### Contour Quality

Expert consensus contours were generated in the following steps (Fig 1). First, three sets of contours were independently made by three head-and-neck expert oncologists (J.G.E., H.P. and P.L.) without access to the AI contours used in this study. These were merged using Simultaneous Truth and Performance Evaluation (STAPLE).^26^ The STAPLE-maps were binarized with a threshold of 0.8. The binary STAPLE structures were then reviewed and corrected for artifacts by an external head-and-neck expert oncologist (J.C.) in consensus with J.G.E. Contouring quality was quantified using the Dice Similarity Coefficient (DSC) and Hausdorff Distance 95th percentile (HD95) between participants' contours and expert consensus contours. Increasing DSC and decreasing HD95 indicate increasing agreement with the expert consensus contours and thus higher contouring quality.

The participants were blinded toward the expert consensus contours throughout the entire study.

### Contouring Time

Contouring time was defined as the time of active contouring (mouse click-and-hold) with any contouring tool. For all contouring interactions, duration in milliseconds, type of interaction, active structure, and participant name were automatically recorded by EduCase. The durations of interactions were summed over structures and are reported in minutes.

### Statistics and Software

Data were assumed to be nonparametric. Therefore, effect sizes were quantified as absolute differences of medians with 95% CIs. This is formatted as: Absolute Difference [CI low; CI high]. CIs were estimated with percentile bootstrapping in 9,999 iterations. Absolute differences are positive when the reference value is the smaller number and vice versa. Data handling was performed in Python 3.9, and DSC and HD95 were calculated using MedPy.^27^ Statistics and bootstrapping were performed with SciPy.^28^

## RESULTS

**FIG 2:**
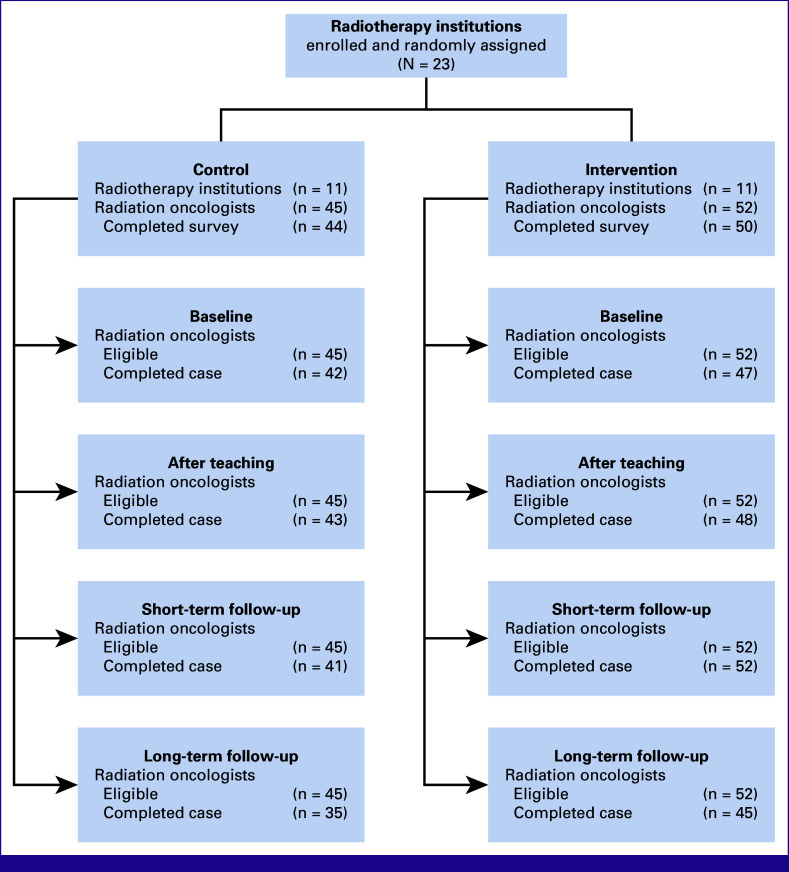
A CONSORT diagram for the study participants. Note that missing one submission did not exclude participants from participating later. Therefore, the number of eligible radiation oncologists is the same throughout all study rounds.

Of the 97 participating ROs, 94 completed the questionnaire on professional background. The random assignment resulted in 11 institutions for the control group and 12 for the intervention group. The characteristics of institutions and participants are found in Table 1. The four rounds of contouring were completed by 89 (92%), 91 (94%), 93 (96%), and 80 (82%) ROs, respectively (Fig 2).

**TABLE 1:** Baseline Characteristics of Enrolled Institution and Participants

Characteristic	Control	Intervention	Total
Institutions, No.			
No. of centers	11	12	23
Participants invited	45	52	97
Annual cases: 21-50	2	3	5
Annual cases: 51-100	2	2	4
Annual cases: 101-200	4	3	7
Annual cases: 201+	3	4	7
Dedicated head-and-neck unit	7	6	13
Use any contouring guidelines	10	11	21
Use any form of auto-contouring	6	6	12
Participants, No.			
Completed questionnaire	44	50	94
Median working years [95% CI]	8 [7 to 12]	8 [4 to 12]	—
Head-and-neck among specialties	13	14	27
Regular user of auto-contouring	20	20	40
No knowledge of auto-contouring	6	7	13
Basic knowledge of auto-contouring	26	29	55
Intermediate knowledge of auto-contouring	9	9	18
Advanced knowledge of auto-contouring	3	5	8
Active in research and development of auto-contouring	6	6	12

**FIG 3:**
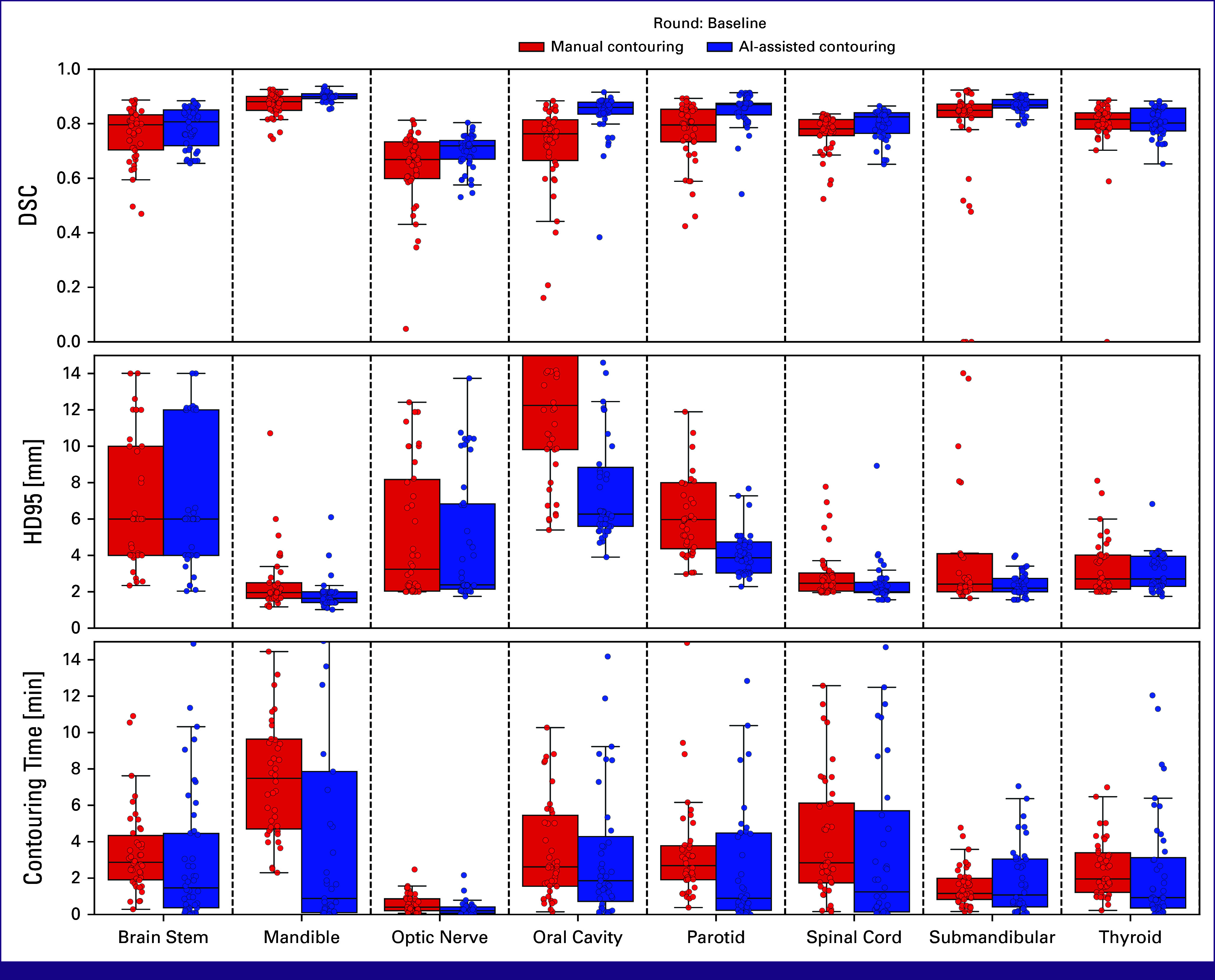
The results at baseline of the two groups. In red is the control group contouring manually, and in blue is the intervention group doing AI-assisted contouring. Each circle represents a single contour from a single participant, and boxplots are based on these. The top and middle rows show DSC and HD95 between participants' contours and expert consensus structures, respectively. The bottom row shows the contouring time recorded by the contouring platform. AI, artificial intelligence; DSC, Dice Similarity Coefficient; HD95, Hausdorff Distance 95th percentile.

**FIG 4:**
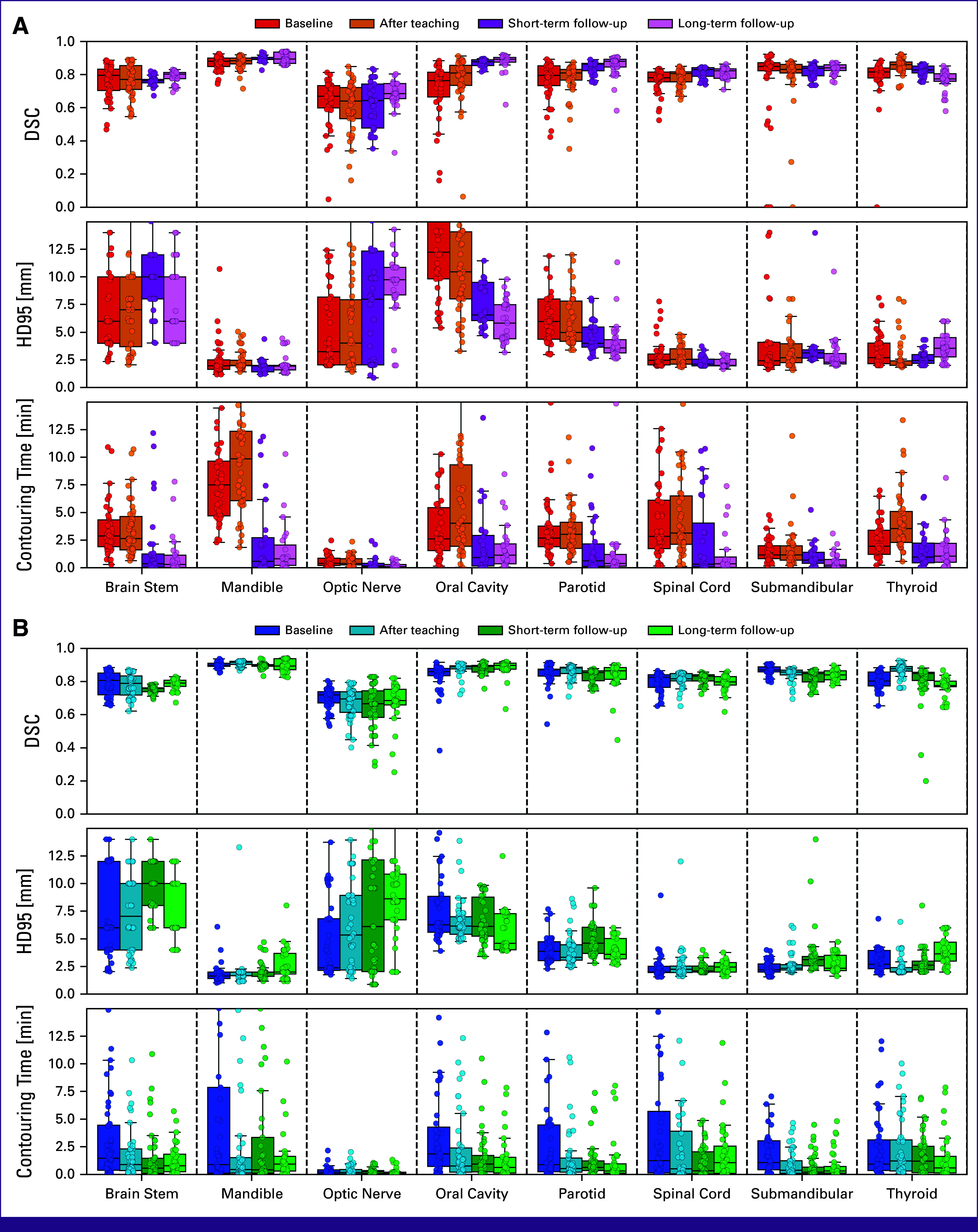
Raw results for the control group (A) and the intervention group (B) for the entire study. Each contouring round is represented by a color. In the control group (A), red is baseline, orange is the round after teaching, dark purple is short-term follow-up, and light purple is long-term follow-up. In the intervention group (B), dark blue is baseline, light blue is after teaching, dark green is short-term follow-up, and light green is long-term follow-up. Each circle represents a single contour from a single participant, and boxplots are based on these. Within each figure, the top and middle rows show DSC and HD95 between participants' contours and expert consensus structures, respectively. Bottom rows show the contouring time. DSC, Dice Similarity Coefficient; HD95, Hausdorff Distance 95th percentile.

**FIG 5:**
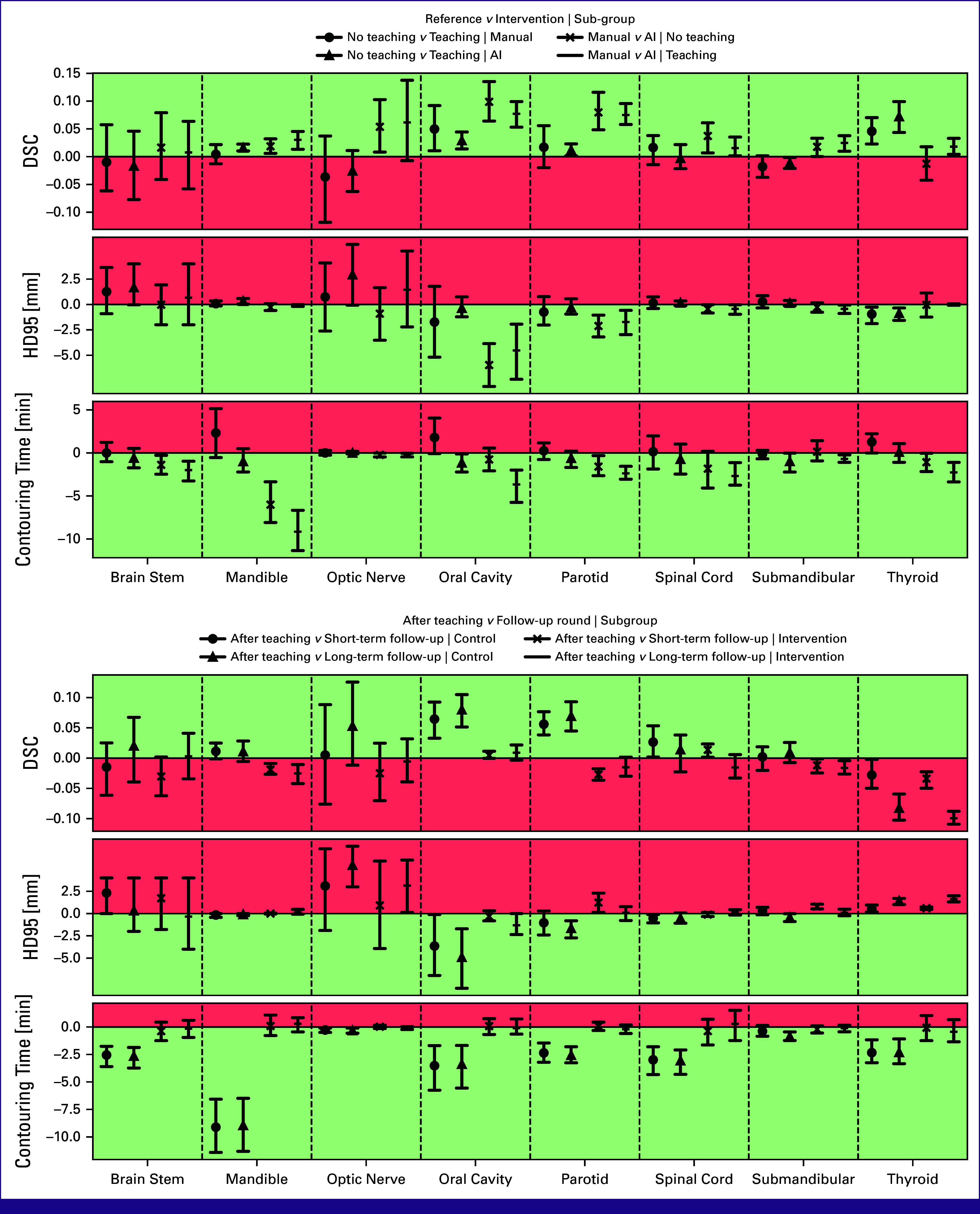
The top figure shows the estimated effect sizes of a given combination of exposures, and the bottom figure shows the estimated effect sizes at short-term follow-up. Top, middle, and bottom rows within each subfigure are DSC, HD95, and contouring time. In the top subfigure, no teaching and no AI-assisted contouring are used as reference in all four subanalyses, and in the bottom subfigure, the round after teaching is reference in all four. Absolute differences of medians are shown with 95% CIs. Values above 0 mean that the reference number is smallest and vice versa. AI, artificial intelligence; DSC, Dice Similarity Coefficient; HD95, Hausdorff Distance 95th percentile.

The raw results of contouring quality and contouring time are shown for each group at baseline in Figure 3 and for all contouring rounds in Figure 4. Below is a walk-through of the estimated effect sizes of the four combinations of exposures along with the effect of combining teaching and AI-assisted contouring in the follow-up rounds (Fig 5). Visual comparisons between manual contouring and AI-assisted contouring are provided in the Data Supplement (Fig S2).

### AI-Assisted Contouring Without Teaching

By comparing the two study groups at baseline (Fig 3), the effect of AI-assisted contouring without teaching was quantified (Fig 5, top, circles). This showed a higher (absolute) median DSC with AI-assisted contouring of 0.05 [0.01; 0.10] (CI) for optic nerve, 0.10 [0.06; 0.13] for oral cavity, 0.07 [0.05; 0.12] for parotid, 0.04 [0.01; 0.06] for spinal cord, and 0.02 [0.01; 0.03] for mandible. Correspondingly lower HD95 was only observed for oral cavity and parotid. Contouring times were lower with AI-assisted contouring by –1.41 [–2.44; –0.25] minutes for brain stem, –6.60 [–8.09; –3.35] minutes for mandible, –0.19 [–0.47; –0.02] minutes for optic nerve, –1.80 [–2.66; –0.32] minutes for parotid, and –1.03 [–2.18; –0.05] minutes for thyroid.

### AI-Assisted Contouring With Teaching

To address the combined effect of teaching and AI-assisted contouring, the two study groups were compared after teaching (Fig 5, top, triangles). The tendency was similar to that of AI-assisted contouring without teaching, with median DSC being higher with AI-assisted contouring by 0.03 [0.01; 0.05] for mandible, 0.05 [–0.01; 0.14] for optic nerve, 0.08 [0.05; 0.10] for oral cavity, 0.07 [0.06; 0.10] for parotid, 0.01 [0.00; 0.04] for spinal cord, 0.03 [0.01; 0.04] for submandibular, and 0.02 [0.00; 0.03] for thyroid. HD95 was only lower for oral cavity and parotid. Contouring time was lower with AI-assisted contouring by –1.76 [–3.26; –0.93] minutes for brain stem, –9.41 [–11.36; –6.71] minutes for mandible, –0.21 [–0.46; –0.04] minutes for optic nerve, –3.28 [–5.74; –1.99] minutes for oral cavity, –2.50 [–3.06; –1.56] minutes for parotid, –2.65 [–4.30; –1.13] minutes for spinal cord, –0.78 [–1.08; –0.24] minutes for submandibular, and –2.35 [–3.39; –1.11] minutes for thyroid.

### Teaching Without AI-Assisted Contouring

The effect of teaching without AI-assisted contouring was quantified by comparing the round after teaching with baseline in the control group (Fig 5, top, crosses). In this comparison, median DSC increased after teaching with 0.05 [0.01; 0.09] for oral cavity and 0.04 [0.02; 0.07] for thyroid. The differences for the remaining OARs were either small or had inconclusive CIs. Contouring time increased after teaching by 2.36 [–0.51; 5.14] minutes for mandible, 1.42 [–0.08; 4.14] minutes for oral cavity, and 1.60 [–0.04; 2.22] minutes for thyroid. Contouring time was unchanged for the remaining OARs.

### Teaching With AI-Assisted Contouring

For AI-assisted contouring, the effect of teaching was quantified by comparing the round after teaching with baseline in the intervention group (Fig 5, top, horizontal bars). For DSC and HD95, the tendency was similar to teaching without AI-assisted contouring, with increased median DSC after teaching of 0.03 [0.01; 0.04] for oral cavity and 0.07 [0.04; 0.10] for thyroid. However, contouring time decreased after teaching with –1.10 [–2.26; –0.10] minutes for oral cavity and with –1.03 [–2.18; 0.05] minutes for thyroid, whereas it was unchanged for the remaining OARs.

### Teaching and AI-Assisted Contouring Over Time

To address whether the effect of teaching combined with AI-assisted contouring persisted over time, the short-term and long-term follow-ups were compared within the study groups with the round after teaching (Fig 5, bottom). For most OARs, the effect sizes were similar in short-term and long-term follow ups, corresponding to a persistent effect over time. However, median DSC was substantially lower for thyroid at long-term follow-up in both study groups.

### Acceptance of AI Contours

Across all submissions with AI-assisted contouring, 335 (24%) AI contours were accepted without editing by a total of 66 (68%) participants. Among these participants, the median number of accepted contours was three per case. Out of the accepted-as-is contours, 100 (30%) contours had a higher DSC than the group average for the specific case and study arm.

## DISCUSSION

This study investigated the effects of teaching and AI-assisted contouring in a large randomized study on ROs from LMICs. The dropout rate was much lower than expected, and therefore, the study was well powered for the research questions. Regardless of contouring method, teaching improved contouring quality for two OARs. Regardless of teaching, AI-assisted contouring increased contouring quality and reduced contouring time for most investigated OARs. The combined effect of teaching and AI-assisted contouring persisted throughout the two follow-up rounds. The study thereby confirms previous research on time savings^29-32^ and reduction in interobserver variability^33^ obtained by auto-contouring.

Previous research has shown that teaching improves contour consistency and quality.^34,35^ It was therefore somewhat surprising that teaching did not affect contour quality more. This is probably due to participants already contouring well at baseline, and thus there was little room for improvement with the applied metrics. This explanation is supported by the fact that oral cavity was one of the structures that improved with teaching in both study groups. Contouring of oral cavity heavily relies on the guideline definitions. Hence, the increase in contouring quality and contouring time in the control group may indicate that teaching in fact was effective, as the participants spent more time contouring higher quality structures. For the intervention group, this manifested itself with an increase in contouring quality and a drop in contouring time, which could be due to AI contours being in high accordance with the contouring guidelines. Therefore, fewer adaptations were required with the participants' updated knowledge. Besides this, it can, however, not be ruled out that (1) a single teaching session is not enough to change contouring practice of the participants, (2) the rotation to new cases after the teaching session could make contouring easier/harder, and (3) the metrics were not sensitive to subtle differences in contours. The results are, however, good news as the high baseline quality may be attributed to the recent years' efforts in teaching programs and implementation of contouring guidelines. Although teaching did not increase contouring quality as much as expected, teaching modified the time savings of AI-assisted contouring; for most OARs, AI-assisted contouring alone reduced contouring time (crosses, Fig 5), but even larger time savings were observed when AI-assisted contouring was combined with teaching (lines, Fig 5). In effect, this means that similar levels of contouring quality were obtained faster with AI-assisted contouring when accompanied by teaching.

Given the inevitable interobserver variability that also exists between experts, the consensus structures were considered to be of the highest quality obtainable. It was therefore quite extraordinary that AI-assisted contouring enabled participants to make structures with higher similarity to the expert consensus contours compared with manual contouring. Although the consensus contours were generated independently of the AI contours, they were in high agreement (Data Supplement, Fig S3). This suggested that AI-assisted contouring may be an effective strategy not only to conform contouring practices between individuals but even facilitate the implementation of—and adherence to—contouring guidelines across countries and continents.

An additional finding was that outliers were effectively eliminated with AI-assisted contouring. With an (arbitrary) threshold in DSC at 0.4, 2.5% of manual contours and only 0.2% of AI-assisted contours were outliers. Although it is unlikely that all outliers would affect radiotherapy treatment, there were five complete geographical misses observed with manual contouring (Data Supplement, Fig S2). These would likely have affected treatment planning, had they been used clinically. The rate of geographical misses with manual contouring in this study hypothetically translates into one organ missed for every 18 patients contoured. This serves as an imperative reminder that peer review of all contours should be routine clinical practice.

From this study, it is clear that AI-assisted contouring is beneficial when the AI-contouring model locates the right structures and provides decent contours. It is, however, known that these models sometimes fail to do so.^20,36^ It remains unknown what effect erroneous AI contours would have on final contours—or, in other words, how wrong AI contours would have to be, before clinicians realize it and fall back on manual contouring. The failure rate is unknown for the commercial model used in this study. Therefore, it cannot be determined whether the benefit of avoided outliers supersedes the risk that may come with erroneous AI contours.

A major limitation to the study was that contours were not used clinically. Most participants completed their cases besides their regular clinical duties, which theoretically increased the risk of automation bias due to the risk of time pressure, lack of interest, and lack of accountability for treatment.^37^ The fact that two thirds of participants handed in all the accepted-as-is-contours suggests either that automation bias was at play for these participants or that these participants might have cognitively processed the review of AI contours differently. The underlying mechanisms of reviewing and editing AI contours are an important topic for future research but are beyond the scope of this work. To confirm the findings of this study, prospective clinical studies on AI-assisted contouring in LMICs should be carried out upon clinical implementation.

ROs who worked in low- and middle-income countries contoured most OARs for head-and-neck cancer with higher similarity to expert consensus contours with AI-assisted contouring than with manual contouring. Furthermore, teaching combined with AI-assisted contouring was the most effective strategy to reduce contouring time. The benefits of teaching combined with AI-assisted contouring persisted after a 6-month follow-up period. Therefore, AI-assisted contouring—especially when combined with teaching—is a promising contribution to reaching optimal utilization of radiotherapy in the present and future. Therefore, a global transition toward AI-assisted contouring with appropriate clinical monitoring is encouraged.
